# Deep clustering of small molecules at large-scale via variational autoencoder embedding and K-means

**DOI:** 10.1186/s12859-022-04667-1

**Published:** 2022-04-15

**Authors:** Hamid Hadipour, Chengyou Liu, Rebecca Davis, Silvia T. Cardona, Pingzhao Hu

**Affiliations:** 1 Department of Computer Science, University of Manitoba, Winnipeg, MB Canada; 2 Department of Electrical and Computer Engineering, University of Manitoba, Winnipeg, MB Canada; 3 Department of Chemistry, University of Manitoba, Winnipeg, MB Canada; 4 Department of Microbiology, University of Manitoba, Winnipeg, MB Canada; 5 Department of Medical Microbiology and Infectious Diseases, University of Manitoba, Winnipeg, MB Canada; 6 Department of Biochemistry and Medical Genetics, University of Manitoba, Room 308 - Basic Medical Sciences Building, 745 Bannatyne Avenue, Winnipeg, MB R3E 0J9 Canada; 7 CancerCaree Manitoba Research Institute, CancerCare Manitoba, Winnipeg, MB Canada

**Keywords:** Unsupervised deep clustering, K-means, Embedding, Variational autoencoders, Internal clustering measurements, Chemical diversity

## Abstract

**Background:**

Converting molecules into computer-interpretable features with rich molecular information is a core problem of data-driven machine learning applications in chemical and drug-related tasks. Generally speaking, there are global and local features to represent a given molecule. As most algorithms have been developed based on one type of feature, a remaining bottleneck is to combine both feature sets for advanced molecule-based machine learning analysis. Here, we explored a novel analytical framework to make embeddings of the molecular features and apply them in the clustering of a large number of small molecules.

**Results:**

In this novel framework, we first introduced a principal component analysis method encoding the molecule-specific atom and bond information. We then used a variational autoencoder (AE)-based method to make embeddings of the global chemical properties and the local atom and bond features. Next, using the embeddings from the encoded local and global features, we implemented and compared several unsupervised clustering algorithms to group the molecule-specific embeddings. The number of clusters was treated as a hyper-parameter and determined by the Silhouette method. Finally, we evaluated the corresponding results using three internal indices. Applying the analysis framework to a large chemical library of more than 47,000 molecules, we successfully identified 50 molecular clusters using the K-means method with 32 embeddings based on the AE method. We visualized the clustering result via t-SNE for the overall distribution of molecules and the similarity maps for the structural analysis of randomly selected cluster-specific molecules.

**Conclusions:**

This study developed a novel analytical framework that comprises a feature engineering scheme for molecule-specific atomic and bonding features and a deep learning-based embedding strategy for different molecular features. By applying the identified embeddings, we show their usefulness for clustering a large molecule dataset. Our novel analytic algorithms can be applied to any virtual library of chemical compounds with diverse molecular structures. Hence, these tools have the potential of optimizing drug discovery, as they can decrease the number of compounds to be screened in any drug screening campaign.

## Introduction

In the practice of drug discovery, high-throughput screening (HTS) is the primary approach for identifying drug candidates from chemical libraries [1]. Nevertheless, screening is an expensive and time-consuming process, especially with the emergence of multidrug-resistant and extensively drug-resistant infections, which create formidable obstacles and challenges for this conventional drug discovery pipeline. To this end, various machine learning (ML) models have been developed and integrated as part of routine protocols in chemical and biological applications for decades [2]. For instance, quantitative structure-activity relationships (QSAR) and quantitative structure-property relationships (QSPR) models played a major role in molecular property predictions, one of the central tasks in drug discovery [3–5]. On the other hand, unsupervised ML methods have been extensively applied in the contexts of exploring molecular data sets and discovering the underlying molecular mechanisms of action (MOA) of new drugs [6]. To establish an efficient ML model for chemical-related tasks, two core questions need to be answered: (1) how to encode a molecule in a machine-interpretable representation with the inclusion of informative and unique features of compounds (molecular featurization); (2) How to ensure the molecular database is diverse enough so that a ML model can learn sufficient chemical patterns to predict the desired properties outside of the training data.

In general, molecular representations can be divided into two main categories: chemical descriptors or fingerprints and representations that are aggregated from molecular graphs [7]. Chemical descriptors and fingerprints are deterministic characterizations of molecules in cheminformatics, and they are commonly employed as the input of conventional QSPR/QSAR models. For instance, extended-connectivity fingerprints (ECFP), a type of topological fingerprints that characterize molecular structures through circular atom neighborhoods, are wildly adopted in QSPR/QSAR models [8]. On the other hand, a molecular graph is a non-Euclidean structural representation composed of a set of atoms (*V*) and a set of chemical bonds or interactions (*E*) between each pair of adjacent atoms [9]. In principle, the molecular graph can be treated as a connected undirected graph *G* defined by a set of nodes (*V*) and edges (*E*). In practice, various chemical properties can be calculated for each atom/bond (local features) so that a molecular graph is initialized by an atomic feature matrix ($x_{v}$) and a bond feature matrix ($e_{vw}$). To utilize local features of molecules for cheminformatics tasks such as molecule property prediction or clustering, the atomic and bond features need to be aggregated to the molecular level.

Clustering is an unsupervised strategy that discovers the existing patterns in a given dataset and classifies the objects into similar groups [10]. In bioinformatics, various clustering algorithms have been implemented depending on different tasks and data [11, 12]. There are three reasons why clustering analysis of compounds in a virtual chemical database must be carried out before developing a QSPR/QSAR model. First, as the quality of predictions from a data-driven model is largely determined by the dataset, validating the diversity of compounds in the selected virtual library ensures that the model learns sufficient chemical information and makes decent predictions. Second, by identifying the similarity or heterogeneity among the chemicals in the dataset, a more comprehensive understanding of drugs' underlying mechanism of action (MOA) could be gained. Finally, clustering analysis can broaden the selection of compounds facilitating the challenging and costly process of establishing datasets for chemical-based ML tasks [4]. Knowing the categories of chemicals that need to be included in the dataset can greatly reduce the number of molecules that should be screened in the laboratory while, at the same time, ensuring the quality of the dataset for the model building.

In this study, we developed a novel molecular embedding learning approach that combines both principal component analysis (PCA) [13] and a variational autoencoder (VAE) [14] to integrate molecular global and local features. We used this approach to cluster ~ 50,000 chemicals previously selected for a large-scale chemical-genetic screen against the bacterium *Mycobacterium tuberculosis* [15], where chemical-genetic interaction profiles (CGIP) were created using *M. tuberculosis* mutant strains (hypomorphs). This work provides an in-depth analysis of a large-scale chemical library successfully used to find potential antibacterial activity. Moreover, by investigating the generated compound clusters, we highlight the importance of feature engineering and gain insight into clusters of compounds that may target the same biological systems and thus may possess similar biological functions.

## Results

### Estimating the number of clusters using the integration of local and global features

**Fig. 1:**
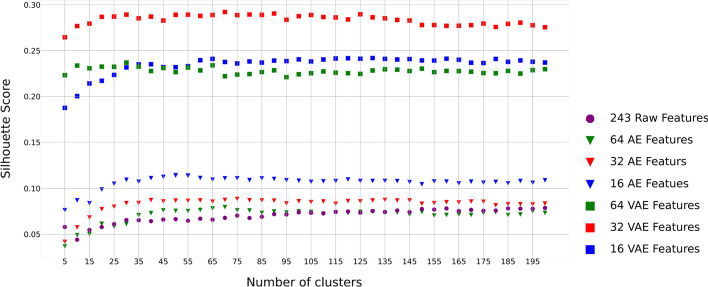
Embedding-specific silhouette scores under different number of molecule clusters using the integration of local and global features. Results are based on the 243 features and the 64, 32, and 16 embeddings from the VAE and AE algorithms, respectively

Using a range of 5–200 clusters with a step size of 5 and different numbers of embeddings (16, 32 and 64) from the autoencoder (AE) [16] and VAE algorithms, respectively, we applied the Silhouette method [17] to estimate the Silhouette scores for the integration of global and local features (Fig. 1). As shown in Fig. 1, all the feature sets or embeddings achieve relatively stable Silhouette scores at a cluster size of 50. Using the 243 integrated local and global features (see details in “Materials and methods” section) produced the lowest Silhouette value, while the best embeddings are the 32 latent features from the VAE algorithm with the largest Silhouette value 0.286 at the cluster size 50 (Fig. 1).

### Performance evaluation of the identified molecular clusters using the integration of local and global features

Table 1 summarizes and compares the clustering performance of the four suggested algorithms (K-means [18], BIRCH (Balanced iterative reducing and clustering using hierarchies) [19], AE + K-means and VAE + K-means) based on the 243 integrated local and global features and their embeddings of the molecule data set from AE and VAE, respectively. For the K-means and BIRCH, we determined the optimal number of clusters 30 based on the 243 features (Fig. 1). For AE + K-means and VAE + K-means, we determined the optimal number of clusters based on different numbers of embeddings (16, 32, and 64) (Fig. 1, Table 1). Overall, based on the three internal measurement indexes, we found the algorithm of VAE + K-means with 32 embeddings showed the best performance (Calinski–Harabasz Index [20]: 10112.928, Silhouette Index: 0.286, and Davies–Bouldin Index [21]: 0.999) with 50 optimized clusters while K-means and BIRCH with the 243 features showed the worst performance (Table 1).

**Table 1:** Clustering performance evaluation using the integration of local and global features

Clustering method	#Clusters	Internal indices		
		Calinski–Harabasz	Silhouette	Davies–Bouldin
K-means	30	1010.383	0.066	2.167
BIRCH	30	825.288	0.042	1.964
VAE (16) + K-means	50	5545.491	0.236	1.142
VAE (32) + K-means	50	**10,112.928**	**0.286**	**0.999**
VAE (64) + K-means	70	4965.177	0.229	1.183
AE (16) + K-means	50	1498.595	0.116	1.703
AE (32) + K-means	40	1117.688	0.085	1.912
AE (64) + K-means	70	717.636	0.075	2.260

The best result of each performance index is boldfaced

### Comparison of clustering performance using only local features and only global features

#### Clustering performance using only local features

**Fig. 2:**
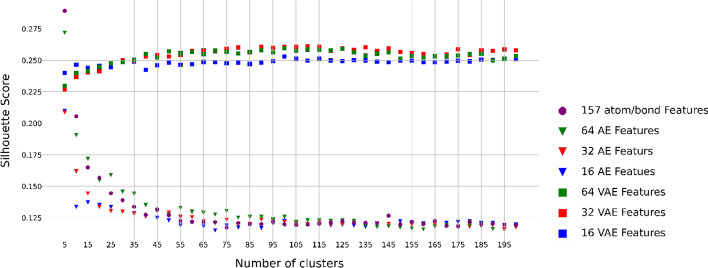
Embedding-specific silhouette scores under different number of molecule clusters using the local features. Results based on the 157 local features and their 64, 32, and 16 embeddings from the VAE and AE algorithms, respectively

Following the same procedure as we did for the integration of local and global features, we applied the Silhouette method to estimate the Silhouette scores for a range of 5–200 clusters with a step size of 5 and different numbers of embeddings from the AE and VAE algorithms using the 157 atomic and bond features, respectively (Fig. 2).

Table 2 summarizes and compares the clustering performance of the four suggested algorithms based on the 157 local features and their embeddings of the molecule data set from AE and VAE, respectively. For the K-means and BIRCH, we determined the optimal number of clusters 55 based on the 157 local features (Fig. 2). For AE + K-means and VAE + K-means, we determined the optimal number of clusters based on different numbers of embeddings (16, 32, and 64) (Fig. 2, Table 2). Overall, based on the three internal measurement indexes, we found the algorithm of VAE + K-means with 64 embeddings showed the best performance (Calinski–Harabasz Index: 9348.354, Silhouette Index: 0.253, and Davies–Bouldin Index: 1.018) with 35 optimized clusters while BIRCH with the 157 local features showed the worst performance (Table 2).

**Table 2:** Clustering performance evaluation using the 157 local features

Clustering method	#Clusters	Internal indices		
		Calinski–Harabasz	Silhouette	Davies–Bouldin
K-means	55	8509.651	0.124	1.704
BIRCH	55	7243.245	0.082	1.831
VAE (16) + K-means	105	7248.059	0.249	**1.007**
VAE (32) + K-means	40	5166.671	0.197	1.194
VAE (64) + K-means	35	**9348.354**	**0.253**	1.018
AE (16) + K-means	30	4666.621	0.145	1.579
AE (32) + K-means	50	5032.735	0.128	1.608
AE (64) + K-means	50	5889.523	0.132	1.626

The best result of each performance index is boldfaced

#### Clustering performance using only the global features

**Fig. 3:**
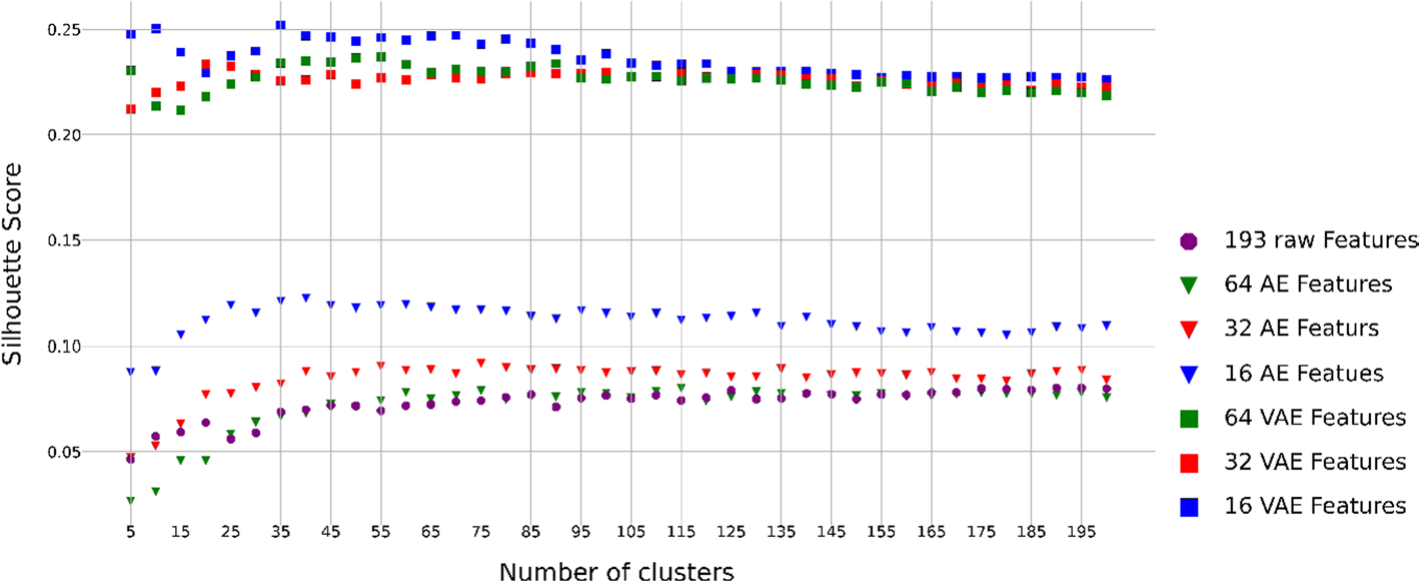
Embedding-specific silhouette scores under different number of molecule clusters using the global features. Results are based on the 193 global features and their 64, 32, and 16 embeddings from the VAE and AE algorithms, respectively. It should be noted that 7 raw global features with low variation were filtered out (see “Materials and methods” section)

Similarly, we also applied the Silhouette method to estimate the Silhouette scores for a range of 5–200 clusters with a step size of 5 and different numbers of embeddings (16, 32 and 64) from the AE and VAE algorithms using the 193 raw global features, respectively (Fig. 3).

Table 3 summarizes and compares the clustering performance of the four suggested algorithms based on the 193 raw global features and their embeddings of the molecule data set from AE and VAE, respectively. For the K-means and BIRCH, we determined the optimal number of clusters 60 based on the 193 raw global features (Fig. 3). For AE + K-means and VAE + K-means, we determined the optimal number of clusters based on different numbers of embeddings (16, 32, and 64) (Fig. 3, Table 3). Overall, based on the three internal measurement indexes, we found the algorithm of VAE + K-means showed the relatively better performance than other methods, while BIRCH with the 193 raw global features showed a relatively worse performance than other methods (Table 3).

**Table 3:** Clustering performance evaluation using the 193 raw global features

Clustering method	#Clusters	Internal indices		
		Calinski–Harabasz	Silhouette	Davies–Bouldin
K-means (193 molecular features)	60	749.187	0.068	1.888
BIRCH (193 molecular features)	60	706.192	0.059	1.710
VAE (16) + K-means	95	4985.544	**0.236**	1.141
VAE (32) + K-means	55	**5168.007**	0.223	1.160
VAE (64) + K-means	65	4991.844	0.227	**1.130**
AE (16) + K-means	45	878.878	0.073	2.009
AE (32) + K-means	45	1112.495	0.090	1.923
AE (64) + K-means	45	1700.526	0.117	1.688

The best result of each performance index is boldfaced

Overall, comparing the performance using only local features (Fig. 2 and Table 2), only global features (Fig. 3 and Table 3) and the integration of local and global features (Fig. 1 and Table 1), it is evident that the algorithm of VAE + K-means with 32 embeddings based on the integrated local and global features has better performance than those of different models based on only local features and only global features.

### Visualization of the identified clusters from the integrated local and global features

**Fig. 4:**
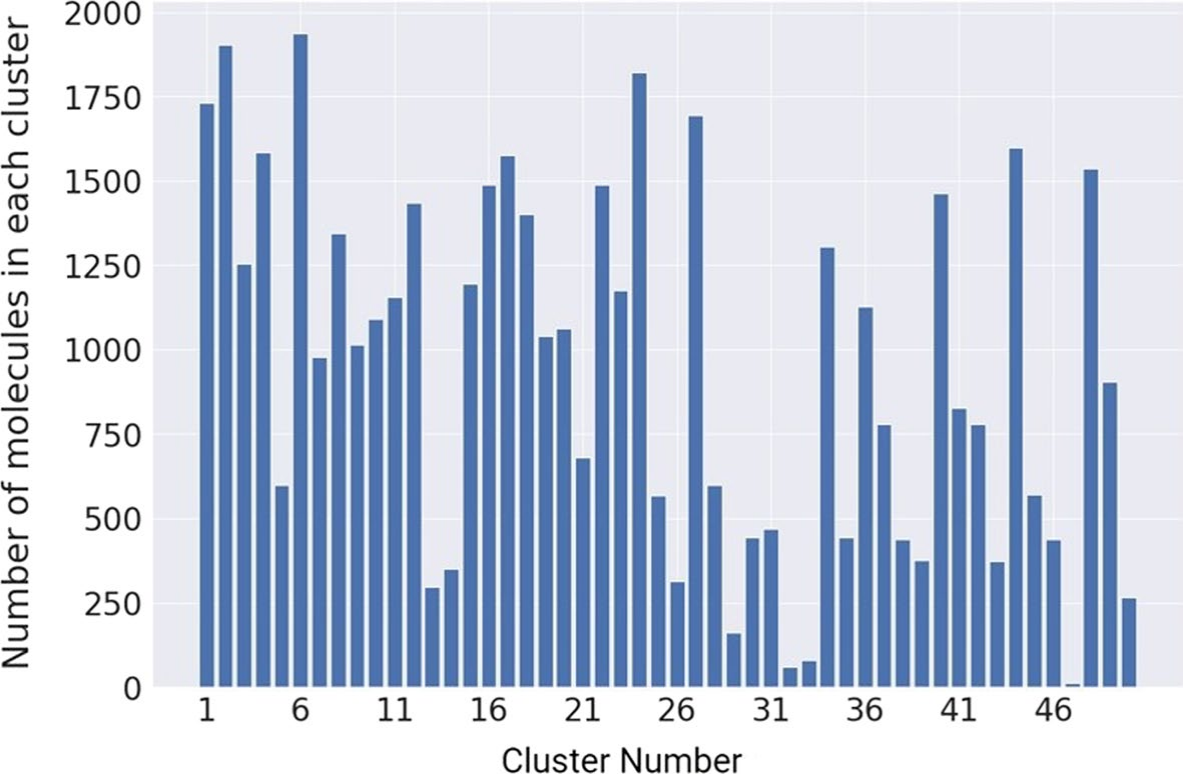
Distribution of molecules in each cluster

We evaluated the distribution of the molecules in each cluster based on the number of molecules using the results from the VAE-based K-means clustering with 32 embeddings (VAE (32) + K-Means) and 50 clusters (Fig. 4). The results are based on the integrated local and global features. As shown in Fig. 4, more than 80% of the clusters with more than 500 molecules and the cluster size is relatively homogeneous.

**Fig. 5:**
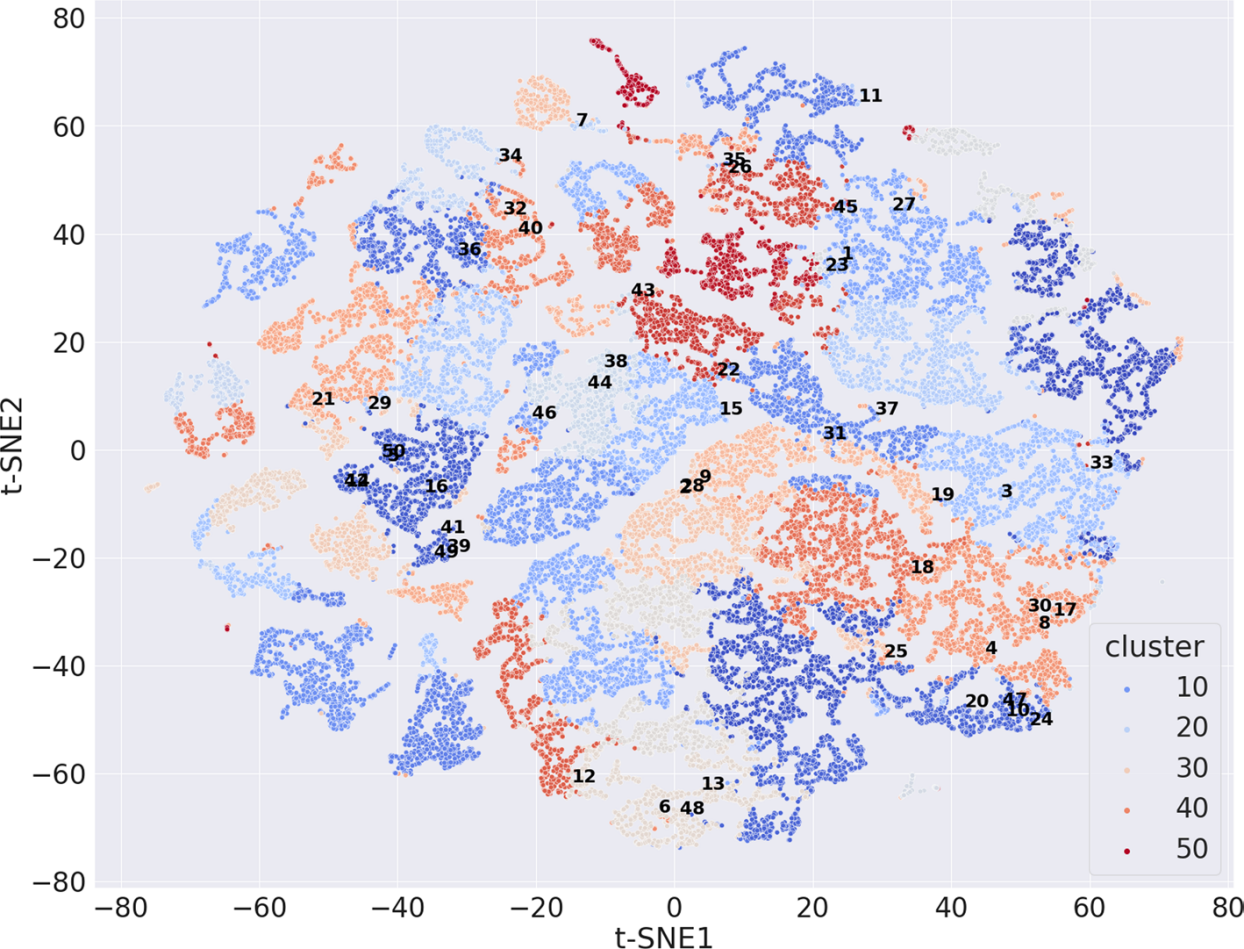
The t-SNE visualization of the 32 embeddings from the VAE algorithm. The numbers are the cluster IDs from the results of VAE (32) + K-means. The colors represent the t-SNE analysis results

Furthermore, we visualized the embeddings from the results with the best algorithm (VAE (32) + K-means) using the t-SNE method [22] (Fig. 5). Overall, the clustered molecules using the VAE (32) + K-means with 50 clusters showed consistent patterns with the t-SNE analysis of the embeddings. The t-SNE clustered the majority of the cluster-specific molecules from the VAE (32) + K-means together.

**Fig. 6.**
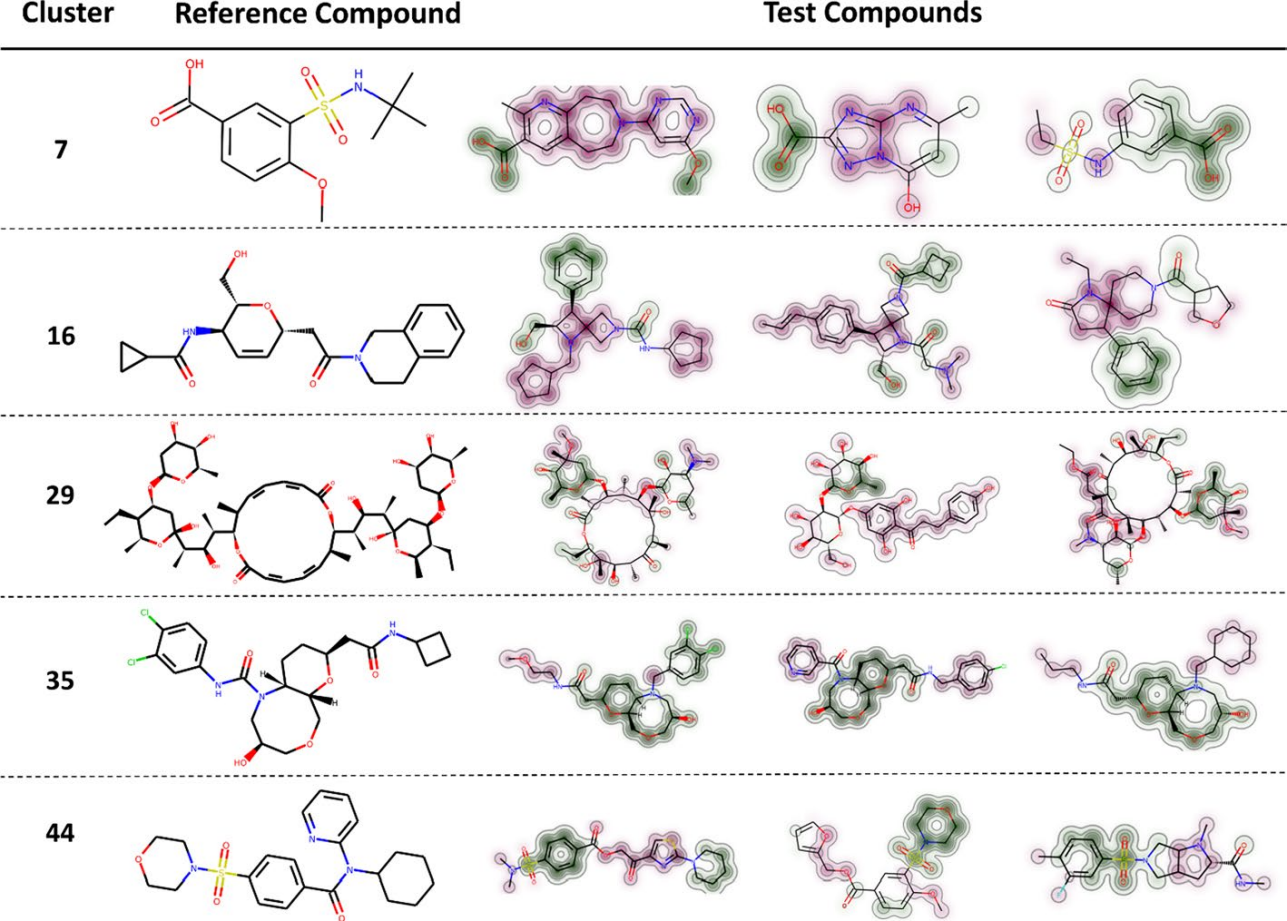
2D structure and similarity map for the examples randomly selected in the five clusters. For each cluster, the similarity scores between the reference compound and three test compounds were measured by the Tanimoto metric using the count-based ECFP (radius = 2, bit = 2048). The similarity weights were visualized by colors on the structure (similarity maps). Sub-structures that increase the similarity score were presented in green, whereas red indicates the opposite

To further examine the effectiveness of our clustering framework and discover the commonalities in molecular structures within the same cluster, four samples, including one reference molecule and three test molecules, were randomly selected from each of five randomly chosen clusters and visualized (Fig. 6). During the generation of the similarity maps [23], the count-based ECFP with radius 2 and 2048 bits was used as the compound representation. In addition, the Tanimoto [24] was selected as the metric during the fingerprint comparison as it is one of the best choices for fingerprint-based similarity calculation reported by Bajusz et al. [25]. In the similarity maps, atoms that contribute to the similarity score between the reference compound and the test compound are highlighted in green, whereas red represents the opposite contribution.

**Fig. 7:**
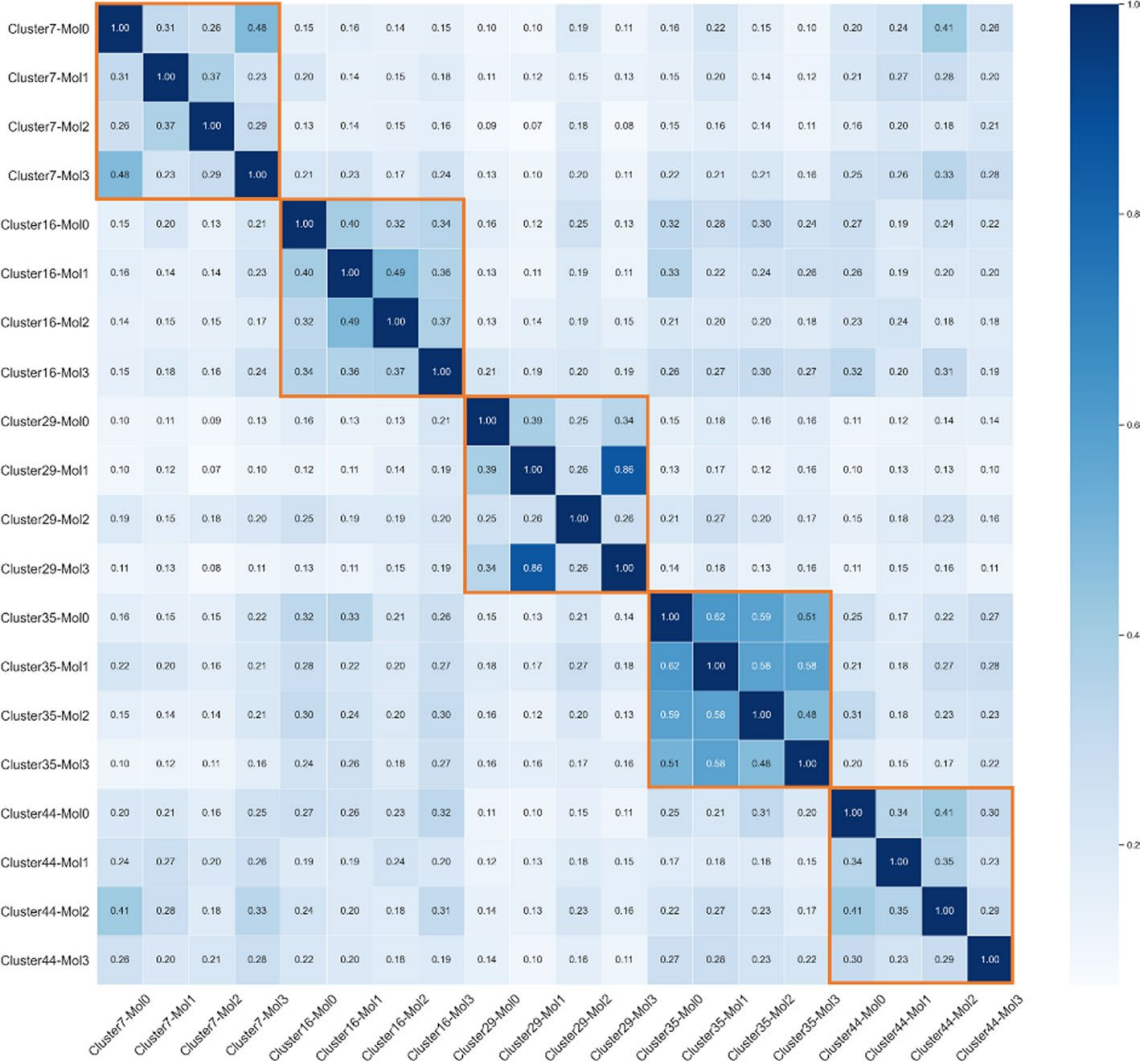
Tanimoto similarity matrix between each pair of the examples, including the reference compounds (Mol0) and three test compounds (Mol1, Mol2, Mol3). The binary ECFP (radius = 1, bit = 2048) were used for the similarity calculation. The orange rectangle circles the samples belonging to the same cluster

From the randomly selected cases in Fig. 6, our clustering framework successfully grouped molecules with more structural similarities into clusters. For instance, all four molecules in cluster 7 contain aromatic carboxylates (labelled in green). Aryl halides appear in three samples in cluster 35, and all samples from cluster 44 contain sulfonamides. We also show the pairwise similarities scores between all selected samples in one matrix (Fig. 7) to present how samples differ within clusters. In order to generate a matrix with a larger contrast, we chose binary ECFP (radius = 1, bit = 2048) as the molecular representation and calculated the Tanimoto score between them. The matrix is diagonally symmetric, and orange rectangles denote samples that belong to the same cluster. The more similar two molecules are, the greater the value of Tanimoto between them. As shown in Fig. 7, it is clear that samples originating from the same cluster obtained larger Tanimoto scores and exhibited darker colors in the matrix. Cluster 35, in particular, has a distinctive difference in color from samples not in this cluster. Mol1 and Mol3 in cluster 29 achieved the highest similarity score (0.86). From their structure in Fig. 6, we can also identify the characteristics of structural closeness between them.

## Discussion

In this study, we first tried to capture molecular descriptors, atomic features and bond features. However, for a given molecule, the molecular descriptor is a feature vector, while the atomic features and bond features are two different matrixes with different dimensions. We explored a simple PCA method to reduce the atomic feature matrix and the bond feature matrix to a PCA-based feature vector, respectively. As the smallest molecule in the Johnson et al. dataset contains only three atoms with two bonds, to simplify the calculation, we only considered the first PC in both the atom and bond-specific PCA, respectively. Generally speaking, the first PC explained at least 60% variance for 80% and 82% of the molecules using the atom features and the bond features, respectively. There is a potential that the number of clusters estimated using different numbers of top PCs from the local bond and atomic features will be different. We will further investigate this interesting question in the future.

It is well known that features normalization is a critical step for creating robust machine learning pipelines, especially for frameworks where distance-based clustering methods such as K-means are used. This is because, for distance-based algorithms, the similarity between each pair of data points is determined by the distances of feature vectors. Thus, the ranges of input features can largely affect the clustering outcome. During our experiments, we did not apply normalization on local features as we have already scaled the atomic mass by multiplying the values with 0.01 and encoded the other atomic and bond features into one-hot numeric arrays. However, for the 200 molecular descriptors generated from RDKit [26], different descriptors have significantly distinct ranges of values, and those with larger absolute values would greatly dominate the clustering process and force the algorithm to be more biased towards them. During our experiments, we found the normalization strategies can significantly impact the clustering results. For instance, we tested both min–max scaling and Z-score scaling individually on each molecular descriptor across all molecules. The Z-score scaling gave the features centered around 0 with a standard deviation of 1. We found this property is especially crucial to compare similarities between features according to the results of three internal metrics.

One major challenge of applying K-means for clustering analysis is to predefine the number of clusters in the data. Here, we applied the widely used Silhouette method to estimate the number of clusters in the large-scale molecule set. However, we expect some other soft K-means methods [27] to perform similar to the methods we applied here. Comparing with the clustering applications in other domains, such as disease subtyping using gene expression profiles, we found that the molecular cluster separation score measured by the Silhouette index is relatively low (the maximum one is 0.286), suggesting the molecules are more diverse and harder to be grouped than data sets from other domains.

As the performance of various unsupervised clustering algorithms (e.g. different variants of K-means) is heavily dependent on the choice of features from the same raw data, much work in this study has been focused on automatically learning these features, or representations of the same raw data. Giving the raw data with 243 integrated local and global features, we explored to autoencoder (use both the standard autoencoder and variational autoencoder) the high dimensional mapping to a lower one with 16, 32 and 64 hidden features, respectively so that the higher dimensional mapping can be reconstructed again. Hence, although the AE(16), AE(32), AE(64), VAE(16),VAE(32),V AE(64) were constructed at different spaces, they are just different set of learned features or representation of the same raw data for K-means clustering. Our deep clustering approach involves two separate processes: one where a representation is learned, and the other where the actual clustering occurs. A better strategy may be to integrate the learning of the representation and clustering into the backward and forward passes of a single learning model so that a single combined loss can be applied. This is an interesting topic we will investigate in the future.

Our results showed that VAE-based embeddings have significantly better performance than AE-based embeddings by performing a simple K-means clustering method on their learned latent vectors. In contrast to the standard AE, which can only construct compact latent representations from inputs and be used as a method of non-linear dimensionality reduction, VAE further generalizes the concept of AE and is able to create new instances by sampling from vectors of means and standard deviations. Given the large-scale dataset used in our study, this additional property of VAE enables the model to generate more accurate and informative latent spaces as the a priori information from the entered molecules gives important control over how a distribution is modelled. Nevertheless, the topological information of molecules is lacking in the latent representation generated from AE/VAE as we only utilized one-dimension (1D) molecular descriptors and local features embedded in 2D space. A potential avenue for future improvement is to incorporate 3D features into the AE/VAE by adding an additional embedding scheme tailored for them so that the topological information of molecules can also be embedded and contribute to clustering.

To summarize, by performing a series of feature aggregation and embedding, we incorporated both global and local features into the clustering analysis of a large-scale compound library and selected the best combination of algorithms (VAE (32) + K-means) as our pipeline according to three internal indices. We investigated the clustering results by calculating the Tanimoto similarities scores of Morgan fingerprints between each pair of randomly selected compounds from five clusters. From the results of the similarity maps, we identified structural similarities within the same clusters and dissimilarities between different clusters. Given the molecular clusters obtained from our framework, it is feasible to carry out the diversity analysis of molecules in each cluster. In addition, based on the molecular properties one wishes to predict, the same QSPR models can be trained on several training sets, which comprise compounds from different combinations of molecular clusters. By investigating the composition of clusters in each training set and their corresponding results on the same test set, we could gain valuable insights into the database itself and the underlying relationships between molecular structures and the desired properties.

## Conclusion

In this study, we developed a novel molecular embedding framework that combines both PCA and VAE to integrate molecules' local and global features. To evaluate the usefulness of the molecular embeddings, we applied our methods to extract the embeddings of the ~ 47,000 molecules from a large-scale molecule library that were screened against *Mycobacterium tuberculosis* mutant strains. We then performed an in-depth clustering analysis of the embeddings by comparing various unsupervised clustering algorithms, including standard K-means, K-means with AE, K-means with VAE, and BIRCH. We demonstrated that embeddings of the molecules using the VAE-based method have significant advantages over those based on the AE-based method. Our analytic framework can be applied to any large-scale chemical libraries with diverse molecular structures. Hence, our novel analytical framework based on the clustering analysis may provide insights for optimizing drug discovery by decreasing the size of screening libraries.

## Materials and methods

### Overall study design

**Fig. 8:**
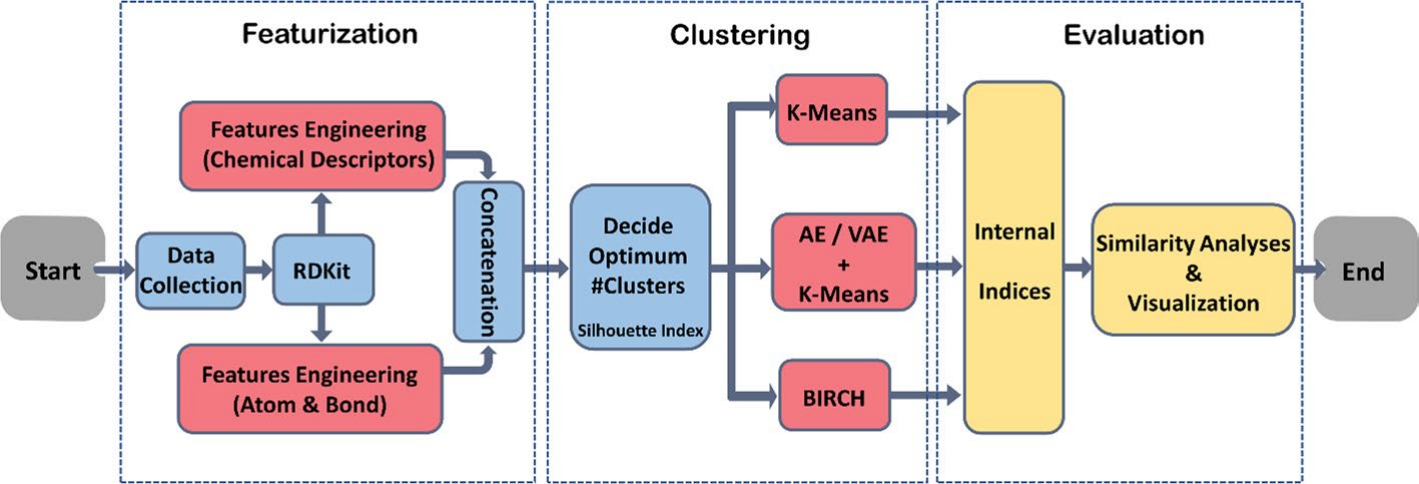
Schematic representation of the study design

The study framework included three parts: molecule featurization, clustering analysis and evaluation (Fig. 8). The first component of our framework is the feature engineering of the compounds. To better take advantage of both the global and local features of molecules, chemical descriptors and atomic and bond features were first generated from RDKit [26]. The atomic and bond feature matrices for each molecule were first summarized and extracted using PCA, then incorporated in the clustering analysis along with the chemical descriptors. With the composite representations of molecules, we selected the optimum number of clusters based on the analysis of the Silhouette method. Next, we investigated three clustering methods using the obtained hyper-parameter: K-means, K-means with autoencoder, and BIRCH. Lastly, we evaluated and compared the clustering methods on three internal indices and visualized examples from five clusters employing similarity maps.

### Data sources

The Johnson et al. [15] dataset used in this study is publicly available on the website (https://www.chemicalgenomicsoftb.com), where the structure and function annotation of 47,217 compounds represented in the simplified molecular-input line-entry system (SMILES) [28] is provided. We used the SMILES strings and the bond and atomic information of the compounds to analyze the distribution and diversity of chemicals.

### Generation of molecular descriptors

A collection of 200 descriptors was derived from different modules in the RDKit package, ranging from basic descriptors such as molecular weight and the number of radical electrons to topochemical descriptors (e.g. Balaban’s J index) and hybrid Estate-VSA descriptors (e.g. MOE VSA descriptors), etc. [29]. The comprehensive cheminformatics descriptors include a wide range of chemical properties at the molecular level, providing a rich source of chemical information on various aspects.

### Generation of atomic and bond features

As defined in the introduction, a molecular graph consists of an atomic matrix ($x_{v}$) and a bond matrix ($e_{vw}$). Table 4 shows the eight types of atomic features and four types of bond features used in this study. All atomic and bond features were one-hot encoded, except for the atomic mass, which was scaled by dividing by 100. Encoding features in a one-hot manner is a common technique for categorical data, which guarantees the algorithm does not consider higher numbers to be more important and allows for a more expressive representation of categorical data [30].

**Table 4:** Descriptions of atomic and bond features

Feature type	Attribute	Size	Description
Atomic features	Atom type	118	Known chemical elements (by atomic number)
	Degree	6	Number of bonds the atom is involved in
	Formal charge	5	Electronic charge assigned to an atom
	Chirality	4	Unspecified, tetrahedral CW/CCW, or other types of chirality
	Number of H	5	Number of bonded hydrogen atoms
	Hybridization	5	sp, sp^2^, sp^3^, sp^3^d, or sp^3^d^2^
	Aromaticity	1	Whether the atom is aromatic
	Atomic mass	1	Mass of the atom
Bond features	Bond type	4	Single, double, triple, or aromatic
	Conjugated	1	Whether the bond is conjugated
	Ring	1	Whether the bond is in a ring
	Stereo	6	Stereochemistry of bonds (none, any, E/Z or cis/trans)

### Feature engineering of molecules

**Fig. 9:**
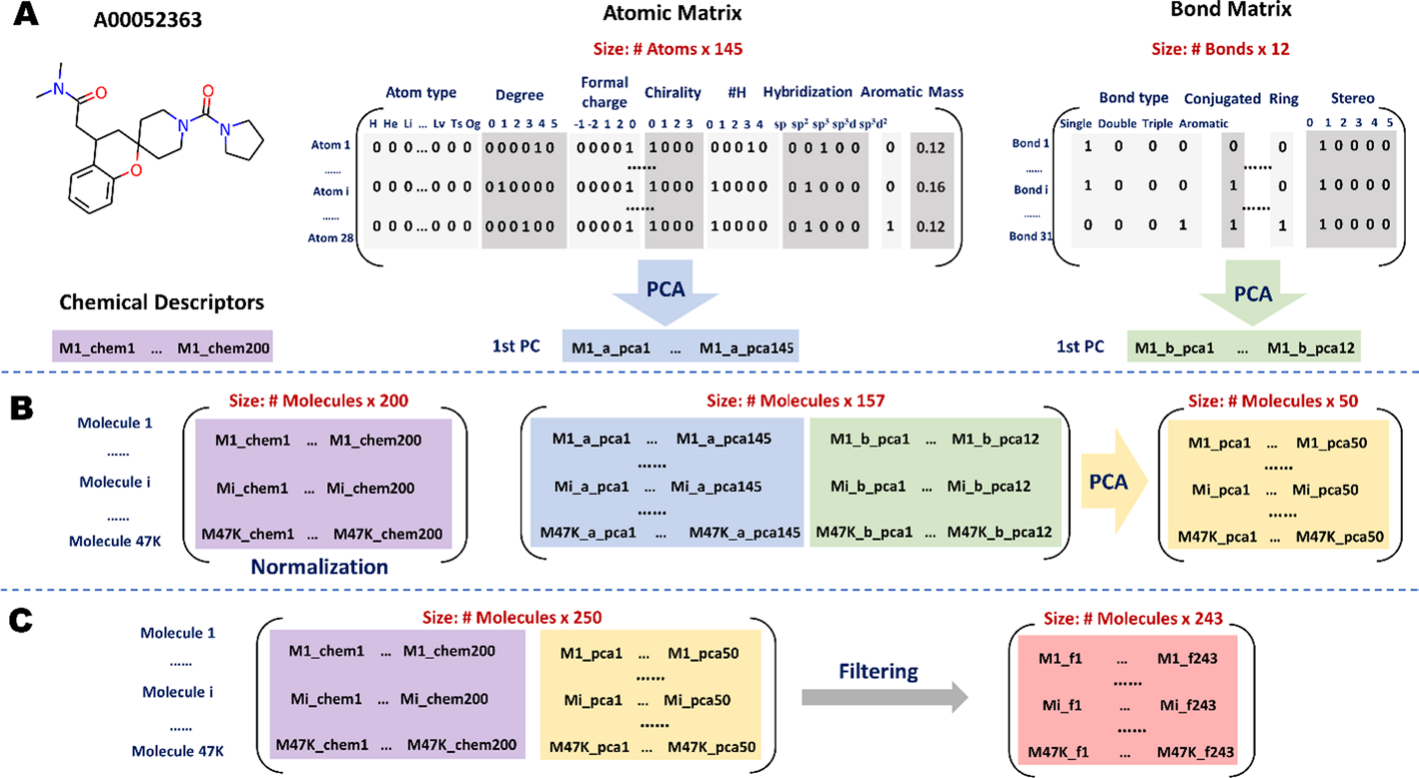
Overview of the feature engineering. **a** The first compound of the Johnson et al. dataset (compound identifier: A00052363) is used as an example. The extracted chemical descriptors and the atomic and bond feature matrices were entered as inputs. Firstly, PCA was performed on each transposed atomic and bond matrix. The first principal component (PC), which contains the greatest amount of variance, was selected as the one-dimensional representation for each feature matrix. **b** The same process was used to iterate through all the compounds in the dataset. We first concatenated the atom and bond features, then performed another PCA on the matrix, and finally selected the top 50 PCAs or features, which explained all the variance in the data. For molecular descriptors, we normalized the values with Z-score scaling among samples. **c** For each molecule, we concatenated the normalized chemical descriptors with the aggregated local features. Finally, we filtered out columns with zero variance, resulting in a feature matrix of size (47,217 × 243) for the subsequent clustering

After extracting the global (molecular descriptors) and local (atomic and bond) features for each molecule, we designed a novel feature engineering scheme (Fig. 9), which fuses the global and local features by performing a series of concatenations and dimensionality reductions.

To utilize graph representations of a molecule, the features of atoms and bonds need to be aggregated and embedded into a vector (readout) for use in subsequent tasks. In this regard, many graph neural networks (GNN) have been proposed, in which molecular features were aggregated via different message passing (or graph convolution) schemes [5, 31, 32]. However, GNNs belong to supervised algorithms, where the ground truth for each molecule is required during training. In other words, the local messages of a molecule can only be updated iteratively via backpropagation on the gradients of the loss between current states and targets. Since we only use the SMILES strings in the library and do not have the ground truth for clustering, we propose a PCA-based approach to combine local molecular features.

PCA is an unsupervised technique of dimensionality reduction that works by finding a new set of mutually uncorrelated variables (principal components) to represent the original data while retaining most of the variation [13, 33]. This study used the linear PCA, which projects the data onto linear subspaces, to aggregate the local features to a lower dimension. Specifically, we performed a linear PCA on each transposed molecule-specific atomic and bond matrix, respectively. The first principal component, which contains the greatest amount of variance, was chosen as the one-dimensional representation of each atomic and bond feature matrix of a given molecule, respectively (Fig. 9a-middle and right panels). In this way, the local features of different sizes in each molecule were aggregated into a representation with the same dimensionality for all molecules (Fig. 9b-middle panel).

**Fig. 10:**
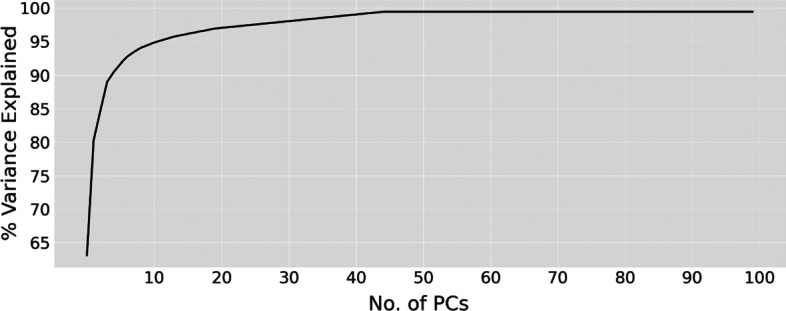
The proportion of variance explained based on the number of PCs. The first 50 PCs explained 100% of the variance of the data

**Fig. 11:**
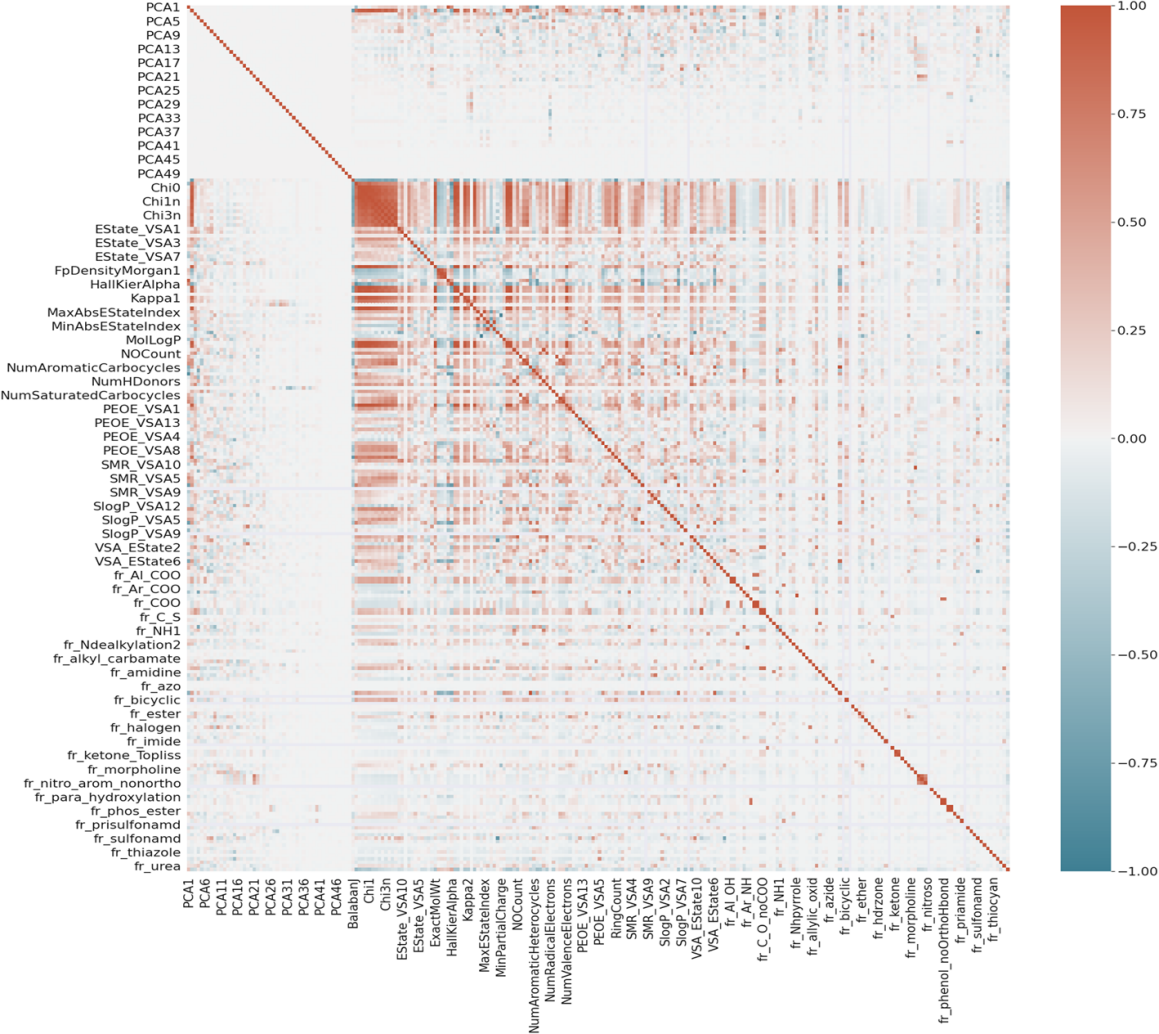
Heatmap showing the correlations between each pair of the features. The 50 aggregated atomic and bond features are named as PCA (1–50), while the rest are the names of the molecular descriptors generated by RDKit

To further filter out the redundant features with low variance across the molecules, we performed another PCA on the concatenated atomic and bond feature matrix (Fig. 9b-middle panel) and selected the top 50 PCAs or features (Fig. 9b-right panel), which explained all of the variance (Fig. 10). To prevent features with larger absolute values from dominating the algorithms, we performed a Z-score normalization of the molecular descriptors so that the values all fell within the same range. Lastly, we concatenated the resulting local and global features, followed by a filtering operation that deletes the feature columns with zero variance (Fig. 9c). The final representation of a molecule is in size of 243 learned features, which incorporate abundant local and global information for the subsequent clustering of the molecules (Fig. 11).

### Molecule clustering

Due to the large number of small molecules in the dataset, we selected below four clustering methods since they are scalable for very large datasets, perform data reduction, and are efficient in memory and time usage.

#### K-means method

K-means [18] is one of the simplest and most famous algorithms used to group objects. K-means starts to indicate centroids (a centroid is the center of a cluster of molecules) randomly. For example, a molecule can be assigned to a particular cluster if it is closer to its centroid than any other centroids. K-means iteratively optimizes the centroids by alternating between assigning molecules to clusters based on the current centroids and choosing centroids based on the current clusters of the molecules. The distances between a given molecule and the centroids are measured by the Euclidean distance metric. The algorithm stops the iterative procedure when either the centroids have been stabilized or when the prespecified number of iterations has been achieved.

#### BIRCH method

BIRCH (Balanced iterative reducing and clustering using hierarchies) is an unsupervised machine learning algorithm used to cluster particularly large datasets. The basic idea of the algorithm is to generate a small and compact summary of a given large dataset but retain as much information as possible [19]. Hence, each clustering decision is made locally, and it does not require to consider all other molecules and currently existing molecule clusters. Compared with other clustering algorithms, this method can use computing memory more efficiently to cluster large data sets. The distances between a given molecule and other molecules are also measured by the Euclidean distance metric in this method.

#### Deep learning autoencoder-based K-means clustering

An autoencoder (AE) is a type of unsupervised neural network that maps input molecules to generate molecule-specific features for reconstructing the input molecules [16, 34]. An autoencoder includes two parts: (1) The encoder that maps the high-dimensional data into low-dimensional data with the most important latent features; (2) The decoder that uses the reduced set of latent features to reconstruct the original input data.

The autoencoder algorithm makes the embedding of the large molecule-specific feature data and reconstructs it in a lower dimension without losing important information. We used K-means to cluster molecule-specific embeddings and generate molecule clusters, which is expected to have much better performance and capture the cluster labels [16].

#### Deep learning variational autoencoder-based K-means clustering

Although AE is simple, controlling how the latent distribution is modelled can be challenging. A variational autoencoder (VAE) [14] is a type of generative neural network based on an autoencoder that is made from an encoder and a decoder. VAE makes the embedding of the input molecule-specific features to a latent space in a probabilistic manner and reconstructs the input data from the latent space. Hence, VAE makes it more practical and feasible for large-scale data sets, like the set of molecules we analyzed here.

**Fig. 12:**
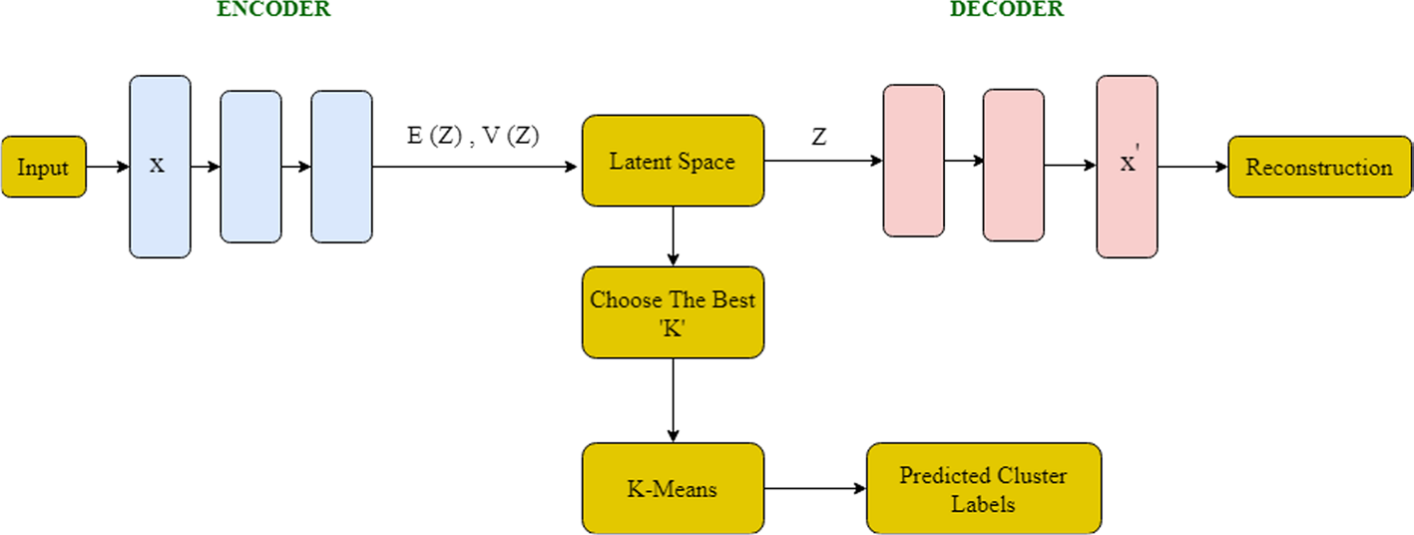
Schematic of the Variational Autoencoder model + K-Means. Encoder: X → R^2d^, Decoder: Z → R^n^. E(Z) represents the mean of the points, and V(Z) is the variance of the points

The general architecture of the VAE algorithm is summarized in Fig. 12. The goal is to minimize the VAE loss that defines as follow,

Reconstruction Loss:

1 $$L = \frac{1}{m}\mathop \sum \limits_{j = 1}^{m} l\left( {x^{j} ,{ }\hat{x}{ }^{j} } \right)$$

where m is the number of molecules, x is the input, and $\hat{x}$ is the output.

VAE Loss:

2 $$L\left( {x,\hat{x}{ }} \right) = l_{reconstruction} + \frac{\beta }{2}\mathop \sum \limits_{i = 1}^{d} \left( {V\left( Z \right) - { }log\left[ {V\left( Z \right)} \right]{ } - 1 + E\left( Z \right)^{2} } \right)_{i}$$

where x is the input data, $\hat{x}$ is the output data, β is the hyperparameter, V (Z) is the variance of the inputs in the encoder section, and E (Z) is the mean of the molecules in the encoder section.

The encoder of the VAE model used in our framework accepts samples of molecule`s features. The encoder contains the combination of six layers of linear, batch normalization layers and an output layer that produces embeddings with reduced-dimension of the samples described above. The decoder subnetwork accepts these encoded samples as input, passing these through an architecture like the encoder, which reconstructs the original samples. In both subnetworks, the activation function of the hidden layers is a ReLu. An Adam optimizer with a learning rate of 1e-3 was used to update the neural networks' weights.

Using the embeddings from the molecule-specific features based on VAE, we applied the K-means algorithm to generate the molecule clusters based on the predefined number of clusters.

### Clustering performance evaluation

After implementing a clustering algorithm, it is necessary to evaluate the quality of the algorithm so that we can choose the clustering algorithm that performs best for an input set of large-scale molecules. Generally speaking, there are external and internal evaluation measures. External evaluation measures usually require a ground truth, which is not available in our study. Hence, we focused on the internal clustering validation. In particular, we applied three widely used performance measures, the Silhouette coefficient [17], the Calinski–Harabasz index [20] and the Davies–Bouldin index [21], to evaluate our clustering performance. The internal clustering measurements were implemented with the “sklearn” python package [35].

#### Silhouette index

Silhouette index [17] is a mathematical method for validating and interpreting the consistency within data clusters. Generally speaking, a simple graphical representation is used to visualize how well each object is grouped. The Silhouette coefficient *s* is expressed as:

3 $$s = \frac{{\left( {b - a} \right)}}{{max \left( {a,b} \right)}}$$

where a is the mean distance between a given molecule and all other molecules in the same cluster, while b is the mean distance between a given molecule and all other molecules in the next nearest cluster. Silhouette coefficient values range between − 1 and + 1, with higher values indicating that the molecules are better clustered. As the Silhouette index is bounded between [− 1, 1] and it indicates the level of cohesion of an object to its own cluster compared to other clusters, it is commonly calculated for finding the optimal number of clusters or used as the validation of consistency within clusters for unsupervised clustering tasks.

#### Calinski–Harabasz index

The Calinski–Harabasz index represents the ratio of the sum of between-clusters dispersion and inter-clusters dispersion of all clusters identified from the analysis [20]. The index can be calculated as a score *S* for *k* clusters:

4 $$S = \frac{{tr\left( {B_{k} } \right)}}{{tr\left( {W_{k} } \right)}} \times \frac{{n_{E} - k}}{k - 1}$$

where the *tr*($B_{k}$) is the trace of the between-group dispersion matrix and *tr*($W_{k}$) is the trace of the within-cluster dispersion matrix. They can be calculated as:

5 $$B_{k} = \mathop \sum \limits_{q = 1}^{k} n_{q} \left( {c_{q} - c_{E} } \right)\left( {c_{q} - c_{E} } \right)^{T}$$

6 $$W_{k} = \mathop \sum \limits_{q = 1}^{k} \mathop \sum \limits_{{x \in C_{q} }} \left( {x - c_{q} } \right)\left( {x - c_{q} } \right)^{T}$$

where *C*_*q*_ is the set of molecules in the cluster *q*. *c*_*q*_ is the center of the cluster *q*. n_*q*_ is the number of molecules in the cluster *q*. *c*_*E*_ is the center of cluster *E*. A higher score indicates a model with more separate clusters.

#### Davies–Bouldin index

The Davies–Bouldin (DB) index is an internal evaluation measure to evaluate the performance of cluster algorithms [21]. It is defined as the similarity of the average between each cluster *Cu*, for *u* = 1, …, *k*, and its most similar one *Cv*. *Ruv* is defined as the similarity given by:

7 $$R_{uv} = \frac{{s_{u} + s_{v} }}{{d_{uv} }}$$

where s_w_ is the diameter of a cluster for *w* = 1, …, *k*; *duv* is the distance between cluster centroids *u* and *v*. The DB index can be calculated as:

8 $$DB = \frac{1}{k}\mathop \sum \limits_{u,v = 1}^{k} maxR_{uv}$$

where a lower DB index means a given model has better separation between the clusters.

Among the three internal performance metrics, the Silhouette index is more commonly used than the other two metrics since its value is bounded between [− 1, 1], which means the value is more interpretable. The main advantage of the Davies–Bouldin index is that it is calculated using only point-to-point distances. Hence, the index is exclusively based on the quantities and features inherent in the data set. In addition, compared to the Silhouette index, the Davies–Bouldin index is simpler to be computed. By definition, the Calinski–Harabasz score is computed quickly and relates to a standard concept of a cluster where a higher score indicates denser and better-separated results.

### Estimation of the number of molecule clusters

One of the major challenges in performing clustering analysis is to decide the number of clusters in a given observed data. One of the most popular methods to calculate this number is the Silhouette index [17]. To do this, we first calculate the Silhouette scores using the observed data under a different predefined number of clusters. We then draw an X–Y plot where the Y-axis is the Silhouette scores, and the X-axis is the different number of clusters. The optimized number of clusters in the observed data is the minimum number of clusters where the Silhouette scores become relatively stable.

### Visualization analysis

#### t-SNE visualization of the molecular embeddings

t-distributed Stochastic Neighbor Embedding (t-SNE) is a statistical tool to visualize high-dimensional data by mapping the data points in high-dimensional space to a two or three-dimensional space in such a way that similar objects (molecules) are modelled by nearby points (molecules) and dissimilar objects are modelled by distant points with high probability [22, 36]. We applied the t-SNE to visualize our embeddings from the VAE analysis.

#### Molecular similarity map

In cheminformatics, a common strategy to quantify the similarity between two compounds is by assessing the fingerprint similarities with distance metrics, such as Dice [37] or Tanimoto [24]. Based on this scheme, the similarity map proposed by Riniker et al. [23] provides the ability to visualize the atomic contribution to the similarity between two molecules or the predicted probability from a given machine learning model. For each atom in a test compound, its atomic contribution (weight) to the similarity to a reference compound equals the similarity difference when the bits in the fingerprint corresponding to the atom are removed. The weights generated for each atom are then normalized and used to color the topography-like map for visualization. We generated the molecular similarity map using the module implemented in the RDKit.

## Data Availability

The raw data from the Johnson et al. study is publicly accessible on the website: https://www.chemicalgenomicsoftb.com/. The scripts, datasets, and results supporting the conclusions of this article are available in the manuscript and our GitHub repository: https://github.com/HamidHadipour/Deep-clustering-of-small-molecules-at-large-scale-via-variational-autoencoder-embedding-and-K-means.

## Abbreviations

PCA: Principal component analysis
AE: Auto encoder
VAE: Variational auto encoder
t-SNE: T-Distributed stochastic neighbor embedding
MOA: Mechanism of action
BIRCH: Balanced iterative reducing and clustering using hierarchies
SMILES: Simplified molecular input line entry system
GNN: Graph neural networks
PC: Principal component
ML: Machine learning
QSPR: Quantitative structure-property relationships
QSAR: Quantitative structure-activity relationships
HTS: High throughput screening
ECFP: Extended connectivity fingerprint
